# Evolutionary concepts in biobanking - the BC BioLibrary

**DOI:** 10.1186/1479-5876-7-95

**Published:** 2009-11-12

**Authors:** Peter H Watson, Janet E Wilson-McManus, Rebecca O Barnes, Sara C Giesz, Adrian Png, Richard G Hegele, Jacquelyn N Brinkman, Ian R Mackenzie, David G Huntsman, Anne Junker, Blake Gilks, Erik Skarsgard, Michael Burgess, Samuel Aparicio, Bruce M McManus

**Affiliations:** 1 Tumour Tissue Repository, Deeley Research Centre, BC Cancer Agency, 2410 Lee Ave, Victoria, BC, Canada; 2 BC BioLibrary, Vancouver, BC, Canada; 3 Department of Pathology and Laboratory Medicine, UBC, Vancouver, BC, Canada; 4 Prevention of Organ Failure Centre of Excellence, Vancouver, BC, Canada; 5 Department of Laboratory Medicine and Pathobiology, Toronto, ON, Canada; 6 The James Hogg iCAPTURE Centre for Cardiovascular and Pulmonary Research, Vancouver, BC, Canada; 7 Department of Pathology, Vancouver General Hospital, Vancouver, BC, Canada Canada; 8 Centre for Translational and Applied Genomics, Vancouver, BC, Canada; 9 Clinical Research, Child & Family Research Institute, Vancouver, BC, Canada; 10 Children's and Women's Health Centre of BC, Vancouver, BC, Canada; 11 Department of Pediatric Surgery, UBC, Vancouver, BC, Canada; 12 College for Interdisciplinary Studies, UBC, Vancouver, BC, Canada; 13 Department of Genetic Pathology, BC Cancer Agency, Vancouver, BC, Canada

## Abstract

**Background:**

Medical research to improve health care faces a major problem in the relatively limited availability of adequately annotated and collected biospecimens. This limitation is creating a growing gap between the pace of scientific advances and successful exploitation of this knowledge. Biobanks are an important conduit for transfer of biospecimens (tissues, blood, body fluids) and related health data to research. They have evolved outside of the historical source of tissue biospecimens, clinical pathology archives. Research biobanks have developed advanced standards, protocols, databases, and mechanisms to interface with researchers seeking biospecimens. However, biobanks are often limited in their capacity and ability to ensure quality in the face of increasing demand. Our strategy to enhance both capacity and quality in research biobanking is to create a new framework that repatriates the activity of biospecimen accrual for biobanks to clinical pathology.

**Methods:**

The British Columbia (BC) BioLibrary is a framework to maximize the accrual of high-quality, annotated biospecimens into biobanks. The BC BioLibrary design primarily encompasses: 1) specialized biospecimen collection units embedded within clinical pathology and linked to a biospecimen distribution system that serves biobanks; 2) a systematic process to connect potential donors with biobanks, and to connect biobanks with consented biospecimens; and 3) interdisciplinary governance and oversight informed by public opinion.

**Results:**

The BC BioLibrary has been embraced by biobanking leaders and translational researchers throughout BC, across multiple health authorities, institutions, and disciplines. An initial pilot network of three Biospecimen Collection Units has been successfully established. In addition, two public deliberation events have been held to obtain input from the public on the BioLibrary and on issues including consent, collection of biospecimens and governance.

**Conclusion:**

The BC BioLibrary framework addresses common issues for clinical pathology, biobanking, and translational research across multiple institutions and clinical and research domains. We anticipate that our framework will lead to enhanced biospecimen accrual capacity and quality, reduced competition between biobanks, and a transparent process for donors that enhances public trust in biobanking.

## Background

In the past decade, unprecedented progress has been made in health research towards realizing the goal of personalized medicine guided by biomarkers and the ability to match the right preventive or treatment with the right patient, at the right time. Key to this progress has been the various '-omics' platforms, as well as bioinformatics, molecular imaging, drug discovery, and in the development of animal models of human disease [1-3]. However, there is now a disparity between the pace of scientific advances and the successful utilization of this knowledge for human benefit. This is partly due to the neglect of a critical platform for this path to personalized medicine - the process of securing biospecimens of the necessary quality, capacity, and level of annotation, and that are truly representative of diseased populations.

### Biobanks

Biobanks are central to the process of collection of human biospecimens for translational research and have contributed to numerous advancements in our understanding and treatment of disease [3,4]. Biobanks are collections of human biospecimens (tissues, blood and body fluids and their derivatives collected for diagnosis and/or for research projects) and their associated clinical and outcome data. These biospecimens are typically obtained from a subset of the public who become patients in the health care system. These patients provide biospecimens during clinic visits, diagnostic or therapeutic procedures, or at autopsy. The biospecimens accrued by biobanks are processed and preserved in a variety of ways to support different clinical and research uses, including fixation, freezing and live cell banking. Annotation encompasses documentation of the biospecimen's composition, as well as linkage to health data associated with the patient and their condition, treatment and outcome. Processed and annotated biospecimens are then released to researchers. This typically occurs through selection of biospecimen cohorts from the biobank database using specified criteria to allow a specific research question to be addressed.

Biobanks range in design and user, from those whose primary focus is to support clinical health care (clinical biobanks, including pathology archives) to those that have evolved to primarily support research. Research biobanks exist in many formats from population biobanks to disease-focused biobanks. The latter include informal biobanks associated with small and large research studies, basic research disease-affiliated banks, and clinical trial-biobanks. An escalating demand for biospecimens is resulting in the transformation of biobanking from an immature 'cottage industry' conducted by individuals, into a complex institutional activity [5,6]. Biobanking has expanded to embrace a range of specialized components including frameworks (ethics, privacy, security), equipment (processing, annotation, storage), operating procedures (biospecimen accrual, processing, annotation, storage, release, distribution, tracking), clinical informatics (pathology, treatment, and outcome data), database structures (donor consent and preference lists, inventory management tools, query tools), policies (priorities and access processes), economic models (funding sources, user fees, intellectual property), governance models (for strategy and operations), and personnel with specialized roles and training. This has meant that research biobanking, which was once an activity mostly limited to clinical pathology, has now evolved largely outside clinical departments as a research discipline. This maturation is also exemplified by the publication of 'Best Practices' by a number of groups [7-10] as well as the development of biobank data infrastructures and common data elements [11-13].

### Bottlenecks in Biobanking

Despite the advances of biobanking described above, significant issues and limitations remain that are restricting the impact of translational research. The major issues include the need to increase the quality and standardization of biospecimens collected, to enhance accrual capacity in terms of scale and disease representation, and above all, to maintain public trust in these activities. Underlying these issues is the need to ensure sustainability of biobanks and to provide mechanisms for equitable and appropriate access to biospecimens.

Quality issues relate to the complications inherent in imposing complex research collection protocols on the routine workflow of distinct clinical organizations. These issues also relate to the difficulty in striking the right balance and appropriate division of biospecimens for both clinical and research requirements ('tissue ethics'). In particular this division makes it difficult to ensure that representative components of the biospecimens exist in both collections. One example of this difficulty is the low frequency with which pre-cancer lesions are captured in research biobanks. Variations between biobanks also influence quality. Even with recent advancement in the way biobanking is conducted, the impact of pre-analytical biospecimen variables, such as collection time [14], is not typically accounted for in translational research.

Capacity issues relate to both geographical and temporal gaps in the biobanking process. The geographic gap occurs because research biobanks have typically developed in health centres with an active research focus, not necessarily those with the highest volume or diversity of surgical and pathology services. Temporal capacity gaps arise because treatment occurs independently of opportunities to engage patients in research. Most biospecimens arise in the course of clinical treatment at a single location and is often completed before the relevance of the biospecimen to research becomes apparent, diminishing the opportunity to harvest biospecimens using specialized research protocols. One example is the patient who chooses to enroll in a clinical cancer therapy trial and has a formalin-fixed paraffin-embedded (FFPE) block created for the clinical archive. The retrieval of the FFPE archival block for a future biomarker assay is often a significant logistic barrier because it has been consigned to the clinical archive several weeks before the patient chooses to become involved in research. Studies requiring a frozen biospecimen are often impossible because retaining a frozen biospecimen is frequently not part of the standard clinical protocol.

Framework issues include inconsistent ethical frameworks, privacy protection efforts and different "business models" between biobanks [15-18]. These issues create uncertainty around accountability to oversight bodies (e.g., ethics boards, privacy offices, and funding agencies) and to the public. This is of particular concern to those who donate their tissue and data to biobanks. These donors have the expectation that their donation will be appropriately, equitably, and maximally utilized to achieve better health care. Events relating to biobanking in the UK provide concrete examples of the effect of failing to address these issues[19,20]. As an activity that spans and directly engages health care, research, and a subset of society, it is essential for biobanking to communicate with these stakeholders and the public at large.

Sustainability issues stem from the nature of funding; the limited scale and the non-systematic resources dedicated to biobanking [21]. It has been the expectation that research biobanks should be able to conform to the business models of other core research technology platforms. Funding for core platforms is typically dependent on local research strengths, dispersed over short durations, and anticipates short-term sustainability or profit. This is clearly at odds with the need to annotate samples with extended outcome information over many years during which clinical practice and research questions evolve to determine the use of specific samples. It is also at odds with the fundamental nature of biospecimens as gifts from generous donors for research. Cost recovery strategies for biospecimen retrieval, processing, and appropriate annotation are emerging but are difficult to deploy in such a way that ensures biobanks are self-sustainable. Thus, ongoing costs of biobanking need to be addressed as this is now an essential component of research translation.

Access issues around biospecimens and their use are seen differently from the perspectives of donors, biobanks, and research users. For donors, it often means having the opportunity to contribute their biospecimen and health data to drive research that can address their specific disease. For biobanks, it means access to potential donors to seek their consent to accrue biospecimens. For research users, it means finding and obtaining the right biospecimens within biobanks and navigating regulatory and oversight processes. Both donors and biobanks face the geographical restrictions noted above, wherein the opportunity to connect and to donate is unavailable due to lack of a formal biobank at the potential donors' health treatment centre. A final issue that contributes to this barrier is the currently pervasive, pre-operative approach/consent paradigm which limits the opportunities for patients to donate to biobanks.

### General Solutions for Biobanking

One solution to address the issues of standardization of quality and capacity is to create networks of biobanks. This idea has stimulated initiatives and networks at regional and national levels including the Canadian Tumour Repository Network [22], CaBIG (cancer Biomedical Informatics Grid) and OBBR (Office of Biorepositories and Biospecimen Research) in the USA [12,23], OnCore in the UK [24], CNIO (Spanish National Cancer Research Centre) in Spain [25], and Biobanking and Biomolecular Resources Research Infrastructure (BBMRI) in Europe [26]. Networks enhance biospecimen and data standards as well as awareness and access by research initiatives [27]. However, networks and associations of biobanks constitute a 'top-down' approach. They do not address local biobanking issues and the geographic and temporal gaps noted above that are critical for quality and capacity in biospecimen and data accrual.

A complimentary strategic solution to networks and associations of biobanks is a 'bottom-up' approach to connect donors and biobanks more effectively. Improved connection between donors and biobanks requires development of processes within health systems to enable potential donors to be referred to biobanks. Currently, many patients are not offered the opportunity to donate to research biobanks despite evidence, including consent rates and donor feedback, demonstrating that this is desirable and beneficial to patients [28]. Although most biobanks do not offer any direct health benefits to the donor, there is thought to be a psychological benefit and a sense of empowerment from donating biospecimens and data to aid scientific and medical advancement [17]. Improvement in the donor-biobank connection requires specific tools to enable donors to register their ongoing status (e.g., disease recurrence and long-term treatment toxicities) and preferences with biobanks. Underlying this is the need for improved connection with the public around the overall activity of biobanking. In the last five years, public awareness of biobanking has grown to the point that it has been ranked as a discipline amongst the top 10 most important ideas that are changing the world [29]. But the public has also been informed that there are associated risks and perils [30], thus progress in biobanking requires public engagement around the governance of the discipline [31].

Improved connections between biospecimens and biobanks requires development of processes to allow biospecimen collection to be conducted in a standardized fashion, responsive to research protocols, and within limits independent of the timing of research consent relative to the time of surgery or therapeutic procedure. This would require re-integration of the biospecimen accrual component of research biobanking into clinical pathology. At the same time, the clinical discipline of pathology needs to adopt processes for maintaining its clinical archives to support the drive to achieve personalized medicine. Assessment of biomarkers are essential for this drive and this is changing the value of the clinical archive from a reference library to a 'real-time' clinical tool [32-35]. Ultimately it might be argued that repatriation of much of current research biobanking to clinical pathology is the best long term approach. This would maintain biospecimens as a valuable resource located within the appropriate privacy environment, facilitate accrual, clinical and histological annotation, and enable appropriate triage for clinical or research purposes to be made on an ongoing basis.

### The BC BioLibrary solution for biobanking

**Figure 1:**
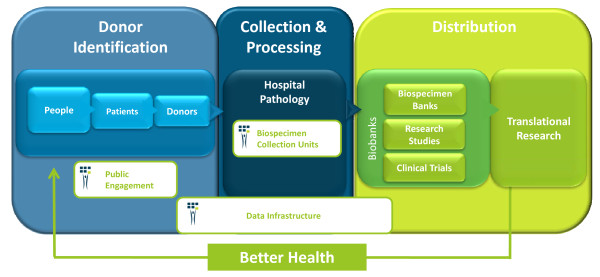
**The BC BioLibrary and its components**. The BC BioLibrary is a framework that lies upstream from biobanks in the cycle that begins and ends with people and leads to their better health. Specifically addressing the aspects of biobanking that involve collection and processing of biospecimens, the components include: 1) the Biospecimen Collection Units which are embedded in the hospital pathology departments and facilitates research orientated biospecimen processing by trained personnel using SOPs; 2) data management infrastructures which enable integration of consent information provided to biobanks with biospecimens from patient donors; and 3) public engagement processes to allow informed deliberation and input from the public into the governance of biobanking.

The British Columbia (BC) BioLibrary http://www.bcbiolibrary.ca is a 'bottom-up' solution and was designed to address issues discussed above. It arose from the desire of a provincial health research foundation (the Michael Smith Foundation for Health Research) to create transformative health research infrastructure to enhance the national and international competitiveness of BC's health research community. A library is defined as a collection of materials organized to provide physical, bibliographic and intellectual access to a target group, with a staff that is trained to provide services and programs related to the information needs of the target group. Thus, a 'biolibrary' is defined as a collection framework that provides all forms of biobanks and their users (translational researchers) with access to human biospecimens. A biolibrary differs from a biobank in that its primary focus is limited to acquisition, cataloguing, and distribution of biospecimens to biobanks (Figure 1). In contrast, a biobank specializes in its capability for biospecimen processing, annotation with histological and donor health data, and long-term storage.

## Methods

### Development of the BC BioLibrary

The BC BioLibrary is a framework which consists of 3 main components: 1) 'Biospecimen Collection Units', established within clinical pathology departments; 2) patient/donor and biobank/user connections and engagement through hospital referral processes and web-based consent and inventory catalogues; and 3) public deliberation to guide its governance. The framework also includes several planned support components including a 'Biospecimen Distribution Unit'. The complete framework as envisaged is described below, followed by the current development status.

### Biospecimen Collection Units

The Biospecimen Collection Units (BCUs) embedded within pathology departments comprise trained biospecimen acquisition personnel (BCU Coordinators) supervised by the appropriate clinical leader within each pathology department. Training provided by the BC BioLibrary and its collection of standard operating procedures extends the skills of pathologists' assistants and technologists with further knowledge surrounding biobanking, research requirements, protocols, ethics and privacy issues. The BCU facilitates the triage of biospecimens into multiple formats, including formalin-fixed paraffin-embedded tissue blocks, flash frozen or OCT-frozen material. Collected biospecimens are held in short term storage and catalogued by logging a unique BC BioLibrary identification number into the relevant clinical pathology record. Elements of this record are extracted into the BCU inventory database (the 'BCU Catalogue').

### Patient/Donor and Biobank Connection

The consent process relating to biospecimen use for research has traditionally involved three distinct steps - permission to contact, the preliminary interview to ascertain interest and preferred medium for detailed discussion, and the informed consent discussion and agreement itself. The BC BioLibrary, acting as an 'honest broker' enables the key first step, by instituting a process to obtain consent after the surgery or therapeutic procedure ('post-operative consent protocol'). The BCU enables pathologists to routinely harvest and hold portions of biospecimens for research, in parallel with the portions of biospecimens sampled and assessed for clinical diagnosis. Once diagnosis has been completed and any immediate diagnostic need for these portions has expired, the consent status and potential research destiny of these research biospecimens can be determined. The BCU facilitates the contact step by communicating with the responsible clinician (the surgeon or their designate such as the medical office assistant) once a potential biospecimen has been harvested, to ascertain if the patient/potential donor will provide permission for contact. If permission is granted, the BCU can forward the referral to the relevant, REB-approved biobank. The biobank can then deploy its own consent protocol or request this service from the BC BioLibrary consent office. Following completion of the consent process, the biobank notifies the BCU Coordinator of the consent status for any biospecimens that have been collected.

**Figure 2:**
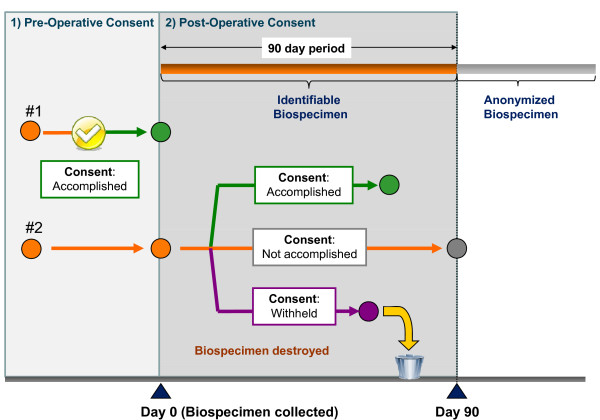
**The possible status of biospecimens collected by the BC BioLibrary BCU, as determined by the consent linked to the biospecimen in relation to the time of surgery**. The consent status of biospecimens collected and held by the BCU is influenced by two possible mechanisms for consent: **#1) Pre-Operative Consent**: If consent is secured pre-operatively by a biobank then the biospecimen (green circle) is collected by the BCU and distributed to the biobank as a coded but identifiable biospecimen that can be linked to the patient donor clinical data by the biobank. **#2) Post-Operative Consent**: If consent is to be sought post-operatively then the biospecimen is collected by the BCU and held as an identifiable biospecimen (orange circle) for a period of up to 90 days (orange lines). During this time the consent status of the biospecimen may change and allow distribution to a biobank as follows: *Accomplished *- biospecimen (green circle) is distributed as per the procedure following a Pre-Operative Consent process. *Not accomplished *- the biospecimen (grey circle) and all related collection data is anonymized and distributed to a biobank (if approved to receive such biospecimens) or destroyed *Withheld *- biospecimen (purple circle) and all related collection data is destroyed.

The status of the biospecimen with respect to the potential donor's specific research interests may already be known through a pre-operative consent process, at the time of harvesting. In this instance the BCU can distribute directly to a specific biobank. If consent has been withheld by the patient the research biospecimen is not collected or is destroyed once this patient decision is known. Alternatively, if the patient has not been approached pre-operatively by a biobank, the biospecimen can be collected held by the BCU for a defined period under an approved post-op consent protocol, before its ability to be used for research is determined. If at the end of the defined period, the consent decision is unknown (e.g., due to inability to make contact with the patient), the biospecimen and all related data are irreversibly anonymized (Figure 2). These anonymized biospecimens may then be distributed to REB-approved biobanks.

### Biospecimen and Biobank Connection through Web-based Consent and Inventory Catalogues

Another key component of the BC BioLibrary is the development of an improved linkage between biospecimens and biobanks via web-based catalogues of existing biospecimens (the 'Biospecimen Inventory Catalogue') and consents (the 'Consent Catalogue').

The Biospecimen Inventory Catalogue component is designed to provide a list of all biospecimens in short-term storage across different BCUs. This component is still under development. It is envisaged that it will be a searchable database for existing biospecimens that are available for distribution from the BCUs or alternatively from biobanks in the community that have an established REB-approved process for request and distribution of their biospecimens. The information available in this database will contain completely anonymized data: the BC BioLibrary ID, donor's age at the time of biospecimen collection, donor's gender, type of biospecimen and disease classification, and its location and availability. Data will be linked to a request form directed to the BC BioLibrary or to the biobank housing the biospecimen.

The Consent Catalogue component will be designed to maintain lists which can be populated by each authenticated, disease-focused biobank seeking access to biospecimens that are collected by the BCUs and that are derived from donors enrolled into the biobank. Access to each list within the Consent Catalogue is restricted to the originating biobank. The Consent Catalogue will be programmed to establish a link between consented donors entered into these lists and their corresponding biospecimens collected in the BCUs. The mechanism for connecting donor consent with the associated biospecimens will be by periodic download of the Consent Catalogue as an encrypted file to each BCU computer workstation. Using an unsupervised query tool, the BCU inventory database will establish linkage between biospecimens at that BCU and consented donors within the Consent Catalogue. All matches will generate a flag in the BCU inventory database as well as a report to enable classification of the biospecimens collected to date by consent status. Based on this report the BCU Coordinator will then destroy, distribute, or anonymize and then distribute biospecimens to the appropriate biobank.

### Public and Biobank Connection through Deliberation

Maintaining and improving public confidence is crucial to the social sustainability of biobanking. Public trust is associated with many topics: governance, clarity of mission and motivation, and transparency around issues of funding and use for academic and industry applications. The BC BioLibrary provides an attractive focus for input from the public on all topics due to its broad scope and direct focus on the primary intersection between patients and biospecimen accrual. The BC BioLibrary has been launched with an initial governance structure designed by biobanking experts and under the external oversight of ethics committees, privacy laws, and health research foundations. However, the intention is to actively seek public input into this structure and to evolve by integrating this input into the oversight of biospecimen collection. Public input is sought through a series of public consultation events and based on a consensus building approach that is fostered by deliberative democracy. The focus of these events will evolve from discussion of general questions around biobanking to more specific discussions around the BC BioLibrary and biobanks and their associated governance models.

### Access to Biospecimens

Access to the BC BioLibrary requires scientific review (conducted by a BC BioLibrary user access committee) to determine priority of each user application and authentication including documentation of research ethics approval (conducted by an institutional REB) to receive and work with the human biospecimens requested. Although still evolving as the BC BioLibrary expands from single site pilot BCUs into a network, the BC BioLibrary user access committee is envisaged to comprise representatives from BCU sites and the BC BioLibrary management and executive teams. The committee conducts scientific peer review scaled to the request and logged through formal applications to assign priority for access to BCUs and seeks to ensure feasibility, fairness and accountability. Single site requests are approved at the local BCU level by the site director, site BCU Coordinator, and the BC BioLibrary manager. External and multi-site requests are handled by the full BC BioLibrary access review committee. All activities are reviewed by the BC BioLibrary Executive. The BC BioLibrary creates a forum to seek resolutions of competing requirements for biospecimens through peer review and draws from collective experience in managing access to biobanks. For those conflicts that persist, a balanced consideration through peer review can help to recognize local priorities while also balancing these with donor preferences and the scientific merit of different projects. Most conflicts can be resolved by shared access, division of the biospecimen, or staggered accrual periods or sites. Another important aspect of user access involves authentication of the users' scientific credentials and the ethical and privacy considerations. REB review and approval addresses these aspects and determines whether access is restricted to biospecimens associated with project-specific consent or can also include anonymized biospecimens.

### Distribution and Backup Storage for Biobanks

Each BCU currently transfers biospecimens direct to the user, but once more BCUs are established, a single portal for transfer and circulation of requested samples (e.g., a centralized 'Biospecimen Distribution Unit') will be more efficient. Users may also choose to receive processed biospecimens and to utilize a range of services and advanced analytical platforms available through the Center for Translational and Advanced Genomics connected to the BC BioLibrary [36]. Once distributed, the ability to properly store and secure frozen biospecimens is the responsibility of biobank users.

## Results

To prepare for initial implementation of the BC BioLibrary plan, we began by delineating the functional components required. A communications plan was developed and a set of key messages derived to articulate components as they related to five overarching goals. The messages were defined as follows: 1) the BC BioLibrary is a facilitator, not a biobank; 2) the BC BioLibrary is intended to help all interested BC researchers and educators; 3) the BC BioLibrary helps pathologists streamline and improve biobanking activities; 4) the BC BioLibrary enhances quality and accessibility of biospecimens; and 5) the BC BioLibrary contributes to the sustainability of biobanking in BC by developing and upholding the public's trust. We pursued this initial 'communication' effort in advance of functional components to reduce the strong potential for misinterpretation of the objectives and motivation underlying a new plan around biospecimen procurement from the many established key stakeholders. The ongoing need to correct the persistent assumption that biobanking can continue as a 'cottage industry' and the misconception that the BC BioLibrary exists to create a single 'BC biobank' underscores the value of this approach.

Implementation began with the establishment of project teams in 2007 to focus on the three main components of the framework: standardization of biospecimens collection and processing ('Biospecimen Collection Unit and Training' team, 13 members); enhanced communication between the donors, biobanks ('Database and Informatics' team, 7 members); and public engagement around biobanking ('Public Engagement' team, 9 members). These teams are managed by an Executive Committee (9 members) and the Management team (3 members), with oversight provided by a Governance Oversight Committee (9 members). Through these teams and committees the BC BioLibrary is driven by leaders in biobanking and translational research across British Columbia, spanning four major academic hospitals, three health authorities, multiple affiliated academic institutions, and five major institutional biobanks. The latter includes the BC Cancer Agency Tumor Tissue Repository (TTR) program [37] and the affiliated TTR Breast Bank, the Ovarian Cancer Research Program of BC [38], the PROOF Centre of Excellence [39], and the James Hogg iCAPTURE Centre[40], as well as many other biobanks embedded within translational research groups.

Each element of the BC BioLibrary has been submitted for REB approval in a stepwise fashion. The first two elements involved establishing a website and a single, pilot BCU in one pathology department. The website served to communicate with stakeholders around all aspects of biobanking and the activities of the BC BioLibrary. Creation of the pilot BCU was essential to provide a working prototype around which we could engage with the REB and pathology stakeholders. To date this first BCU has collected over 450 biospecimens in an 18 month period. Biospecimens collected include those harvested from donors who provided pre-operative consent to two local studies, as well as biospecimens collected under the post-operative consent pilot and not linked to an identified study. The pilot BCU has also been used to develop over 17 SOPs which detail all aspects of biospecimen harvesting and data capture relevant to the BCU, the BCU inventory database ('BCU Catalogue'), as well as a web based training curriculum. The evolution from this single, pilot BCU into a functional accrual network has now begun with the recent establishment of two additional pilot BCUs at additional hospital sites and the graduation of the first pilot to a full BCU approved and capable of supporting multiple biobank users. The two additional web-based Catalogues (Biospecimen Inventory and Consent Catalogues) will be deployed to complete the multi-site biospecimen acquisition capability of the BC BioLibrary.

An important element addressed by the BC BioLibrary is the deployment of a system-wide post-operative consent protocol. The protocol establishes a maximum time span of 90 days from the time of surgery for holding a biospecimen in a BCU. This corresponds to the typical outside limits of the period of completion of the diagnosis. This duration optimally facilitates the necessary clinical process for all biospecimens (pre-surgically consented or otherwise) by enabling portions of the biospecimen to be reclaimed and processed for clinical purposes if necessary to complete the diagnosis. The parallel processes for obtaining permission to contact, completing the consent decision, and assigning consent status to the biospecimen have also been delineated.

The construction of additional components of the framework for centralized distribution has yet to begin. However as part of this planning process the BC BioLibrary conducted a survey in 2008 to gauge the need for frozen biospecimens by BC investigators. The results of this survey showed that over 80% of respondents (n = 55) indicated they were not currently satisfied with their ability to perform their research using biospecimens collected through their own institution. Of those, 98% believed they would benefit from access to biospecimens, with specific requirements for disease-specific (89%) and tissue-specific (77%) biospecimens, collected from more than one institution within the province. The full implementation of the BC BioLibrary BCUs would allow these needs to be met. In addition a literature survey of over 3000 papers reported in cancer research journals at 5 year intervals from 1988 to 2008 shows that use has increased 3 fold. The mean cohort size in research studies that utilized tissue biospecimens has changed from approximately 50 to 150 over this period.

The final and key element addresses public trust. A public engagement process has been launched with the first two events held in 2007 and 2009. The design of these events, the methodology and the composition of the participant groups is described elsewhere [31]. Briefly, the first event involved a diverse group of 25 members of the BC public in an open-ended deliberation on biobanking. Participants were provided with access to information on biobanks and then asked to discuss and share their thoughts, concerns, and perspectives on biobanks. The majority of participants agreed upon support for biobanks in principle and the need for adequate governance of biobanks [31]. The second event built on the first engagement and sought specific input from the public on governance, consent protocols, biospecimen collection, and linkage to health information. In each area, specific questions were considered such as the best person, communication method, and timing for obtaining consent. The outcomes are currently under analysis. The results from the public engagement activities has strengthened our interactions with the Research Ethics Board, physicians and researchers as the public's wishes are in line with the vision of the BC BioLibrary.

## Discussion

Biobanking has historically focused on accrual and annotation of biospecimens, but equally critical is the creation of processes for engaging the public before accrual, distributing biospecimens, and cultivating inter-biobank collaborations. Further efforts towards fostering synergy between the public and biobanks and associated processes will enhance scientific and technological advancement and the translation of discovery to the clinic.

The BC BioLibrary is a novel, province-wide strategy aimed at public engagement in biobanking, a common framework for biospecimen acquisition embedded in pathology departments, and integration of this framework with existing biobanks and a spectrum of research facilities. The design builds on evolutionary concepts including the repatriation of biospecimen acquisition for biobanks back into pathology departments and shared governance of these processes.

As defined above, a 'biolibrary' differs from a biobank. A biolibrary focuses on the complexities of connecting donors with biobanks and on acquisition, cataloguing, and distribution of biospecimens to biobanks. One comparable example of a biolibrary is the Cooperative Human Tissue Network (CHTN) [41]. The program has developed a prospective biospecimen collection system that is linked to a wide variety of individual research and biobank requests. This program is a highly successful framework for support of basic research where the study questions revolve principally around issues that do not require outcome data. The BioLibrary also shares elements with the Shared Pathology Informatics Network (SPIN) [42], designed to enable indexing, annotation and retrieval of biospecimens from clinical pathology archives to certified research projects and investigators. In contrast to the CHTN, this system and its concept was focused principally on archival biospecimens. Both models share design elements with the BioLibrary that 'repatriate' components of biobanking to clinical pathology.

Neither model directly accommodates the consent status of the biospecimen. The CHTN was developed using the non-specific surgical consent as a basis for distribution of anonymized biospecimens with time-of-diagnosis annotation. Both the CHTN and SPIN lack components to effect public engagement. The BC BioLibrary builds on these models to accommodate informed consent status of biospecimens and enable a prospective connection between a biospecimen, the donor's health record, and prospective clinical treatment and outcome data. But perhaps more importantly, the act of communication and the transaction which leads to the approval to collect and store a biospecimen linked to personal health data for research purposes is critical to the future of biobanking. An example of the acute effect on biobanking when public confidence is lost was referred to above [19]. A substantial effort directed at legal and regulatory reform was then required to restore public and government trust and re-enable continued investment in biobanking [20]. Examples of less obvious effects of denying patients the chance to make choices and decisions, and with this the lost opportunity to communicate with them around biobanking, can be gleaned from the study of organ donation rates in countries with opt-in and opt-out systems [43]. The BC BioLibrary framework aims to maximize the opportunities for potential donors to be approached by biobanks for informed consent to participate in research.

Although there is a growing body of evidence for the ethical acceptability of post-operative consent process [44], many biobanks' and their ethics committees have not yet adopted this more attractive approach. By creating a framework that can act as an honest broker, the BC BioLibrary facilitates deployment of a systematic post-operative consent protocol. The BC BioLibrary can therefore overcome geographic gaps for biobanks and facilitate donor opportunities that would not otherwise be possible.

Current regulatory requirements for biobanking have been developed to protect the interests of the public. However, the implementation of regulations to address privacy issues that were developed without biobanking in mind [45] has required adaptation to biobanking processes and poses serious challenges to the pace of research and financial burden to the researchers. At the same time, it is not clear if the range of different interests or the priorities of the public is well served by current regulatory regimes. The establishment of a process of public involvement in parallel with a new process for biospecimen accrual has been essential in gaining trust from professional colleagues around issues such as the motivation of the accrual network and in providing assurance to Research Ethics Boards that the concept and operation of the BioLibrary will be acceptable to the public. Public input, fostered through deliberative democracy events, will help us to devise trustworthy governance and to promote wider public understanding of biobanks [46]. Public involvement will therefore contribute to the social sustainability of the project.

## Conclusion

The BC BioLibrary framework is designed to maximize the opportunity and capability of injecting high quality, accurately annotated biospecimens into all forms of biobanks. This framework addresses geographical and temporal issues that currently limit the capacity and capability of biobanking. In the process, it provides improved opportunity for oversight of biospecimen usage, standardization of consent and collection processes, and equity in biospecimen distribution to biobanks. Perhaps most importantly, by creating a common shared infrastructure, this framework reduces competition between biobanks and offers a transparent process for donors to participate, thereby enhancing public trust and providing an opportunity for public involvement in designing optimal governance of biobanking.

## Abbreviations

BC: British Columbia; BCU: Biospecimen Collection Unit; OCT: Optimal Cutting Temperature compound; REB: Research Ethics Board; SOP: Standard Operating Procedure; MSFHR: Michael Smith Foundation for Health Research.

## Competing interests

The authors declare that they have no competing interests.

## Authors' contributions

The authors' contributions to this manuscript are reflected in the order names are shown. PHW and JEM supervised all aspects of this study and contributed to the manuscript preparation. ROB and SCG participated in the manuscript preparation. All authors contributed to the conception of the ideas embodied here and to the development and implementation of this study. All authors read and approved the final manuscript.

## References

[B1] Towbin JA Bowles NE The failing heart Nature 2002 415 227 233 11805847 10.1038/415227a

[B2] Strausberg RL Simpson AJ Old LJ Riggins GJ Oncogenomics and the development of new cancer therapies Nature 2004 429 469 474 15164073 10.1038/nature02627

[B3] Topol EJ Murray SS Frazer KA The genomics gold rush Jama 2007 298 218 221 17622604 10.1001/jama.298.2.218

[B4] Korn D Commission NBA Contribution of the Human Tissue Archive to the Advancement of Medical Knowledge and the Public Health Research Involving Human Biological Material:Ethical Issues and Policy Guidance 2000 II 1 30

[B5] Morente MM Fernandez PL de Alava E Biobanking: old activity or young discipline? Semin Diagn Pathol 2008 25 317 322 19013897 10.1053/j.semdp.2008.07.007

[B6] Ginsburg GS Burke TW Febbo P Centralized biorepositories for genetic and genomic research Jama 2008 299 1359 1361 18349099 10.1001/jama.299.11.1359

[B7] Dhir R Prostate cancer biobanking Curr Opin Urol 2008 18 309 314 18382241 10.1097/MOU.0b013e3282fb7cbe

[B8] Troyer D Biorepository standards and protocols for collecting, processing, and storing human tissues Methods Mol Biol 2008 441 193 220 18370320 10.1007/978-1-60327-047-2_13

[B9] ISBER 2008 Best Practices for Repositories Collection, Storage, Retrieval and Distribution of Biological Materials for Research CELL PRESERVATION TECHNOLOGY 2008 6 3 58

[B10] US National Institutes of Health-National Cancer Institute National Cancer Institute Best Practices for Biospecimen Resources 2007

[B11] Mohanty SK Mistry AT Amin W Parwani AV Pople AK Schmandt L Winters SB Milliken E Kim P Whelan NB The development and deployment of Common Data Elements for tissue banks for translational research in cancer - an emerging standard based approach for the Mesothelioma Virtual Tissue Bank BMC Cancer 2008 8 91 18397527 10.1186/1471-2407-8-91 PMC2329649

[B12] Welcome to the caBIGTM community Web site https://cabig.nci.nih.gov

[B13] Unified Medical Language System Metathesaurus fact sheet http://www.nlm.nih.gov/pubs/factsheets/umlsmeta.html

[B14] Barnes RO Parisien M Murphy LC Watson PH Influence of evolution in tumor biobanking on the interpretation of translational research Cancer Epidemiol Biomarkers Prev 2008 17 3344 3350 19064549 10.1158/1055-9965.EPI-08-0622

[B15] Auray-Blais C Patenaude J A biobank management model applicable to biomedical research BMC Med Ethics 2006 7 E4 16600040 10.1186/1472-6939-7-4 PMC1475589

[B16] Busby H Biobanks, bioethics and concepts of donated blood in the UK Sociol Health Illn 2006 28 850 865 17184422 10.1111/j.1467-9566.2006.00546.x

[B17] PRIM&R Human Tissue/Specimen Banking Working Group Report of the Public Responsibility in Medicine and Research (PRIM&R) Human Tissue/Specimen Banking Working Group, Part I Assessment and Recommendations 2007

[B18] Gibson E Brazil K Coughlin MD Emerson C Fournier F Schwartz L Szala-Meneok KV Weisbaum KM Willison DJ Who's minding the shop? The role of Canadian research ethics boards in the creation and uses of registries and biobanks BMC Med Ethics 2008 9 17 19014594 10.1186/1472-6939-9-17 PMC2636819

[B19] Hall D Reflecting on Redfern: What can we learn from the Alder Hey story? Arch Dis Child 2001 84 455 456 11369554 10.1136/adc.84.6.455 PMC1718789

[B20] Retained Organs Commission Retained organs Bull Med Ethics 2002 8 11 12408122

[B21] Riegman PH Morente MM Betsou F de Blasio P Geary P the Marble Arch International Working Group on Biobanking for, Research B Biobanking for better healthcare Molecular Oncology 2008 2 213 222 19383342 10.1016/j.molonc.2008.07.004 PMC5527804

[B22] The Canadian Tumour Repository Network https://www.ctrnet.ca/10.1089/bio.2010.8405 24846103

[B23] OBBR Office of Biorepositories and Biospecimen Research http://biospecimens.cancer.gov

[B24] onCore UK http://www.oncoreuk.org

[B25] CNIO http://www.cnio.es/es/index.asp

[B26] Welcome to BBMRI Preparatory Phase http://www.bbmri.eu/

[B27] Riegman PH Dinjens WN Oosterhuis JW Biobanking for interdisciplinary clinical research Pathobiology 2007 74 239 244 17709966 10.1159/000104451

[B28] Malone T Catalano PJ O'Dwyer PJ Giantonio B High rate of consent to bank biologic samples for future research: the Eastern Cooperative Oncology Group experience J Natl Cancer Inst 2002 94 769 771 12011228 10.1093/jnci/94.10.769

[B29] Park A 10 Ideas Changing the World Right Now- #8 Biobanks Time 2009

[B30] The Economist Medicine's new central bankers The Economist 2005

[B31] Secko DM Preto N Niemeyer S Burgess MM Informed consent in biobank research: A deliberative approach to the debate Soc Sci Med 2009 68 781 789 19095337 10.1016/j.socscimed.2008.11.020

[B32] Holland NT Smith MT Eskenazi B Bastaki M Biological sample collection and processing for molecular epidemiological studies Mutat Res 2003 543 217 234 12787814 10.1016/s1383-5742(02)00090-x

[B33] Dowsett M Dunbier AK Emerging biomarkers and new understanding of traditional markers in personalized therapy for breast cancer Clin Cancer Res 2008 14 8019 8026 19088018 10.1158/1078-0432.CCR-08-0974

[B34] Early Breast Cancer Trialists' Collaborative Group Tamoxifen for early breast cancer: an overview of the randomised trials Lancet 1998 351 1451 1467 9605801

[B35] Slamon D Clark GM Wong SG Levin WJ Ullrich A McGuire WL Human breast cancer: correlation of relapse and survival with amplification of the HER-2/neu oncogene Science 1987 235 177 182 3798106 10.1126/science.3798106

[B36] CTAG-Analytical & Laboratory Services http://www.phsa.ca/AgenciesAndServices/Services/PHSA-Labs/About-PHSA-Labs/CTAG.htm

[B37] BC Cancer Agency Tumor Tissue Repository http://bccancer.bc.ca/RES/TTR

[B38] Ovarian Cancer Research Program of BC http://ovcare.ca/research/platforms.php

[B39] PROOF Centre of Excellence http://www.proofcentre.ca/

[B40] James Hogg iCAPTURE Centre Biobank http://www.icapture.ubc.ca/what/what_registry.shtml

[B41] Cooperative Human Tissue Network http://chtn.nci.nih.gov/

[B42] Shared Pathology Informatics Network http://www.cancerdiagnosis.nci.nih.gov/spin/

[B43] Organ Donation Taskforce UK The potential impact of an opt out system for organ donation in the UK: an independent report from the Organ Donation Taskforce 2008

[B44] Hewitt R Watson PH Dhir R Aamodt R Thomas G Mercola D Grizzle WE Morente MM Timing of consent for the research use of surgically removed tissue: is postoperative consenting acceptable? Cancer 2009 115 4 9 19090013 10.1002/cncr.23999

[B45] Key Issues http://www.priv.gc.ca/legislation/02_07_01_01_e.cfm

[B46] Secko DM Burgess M O'Doherty K Perspectives on engaging the public in the ethics of emerging biotechnologies: from salmon to biobanks to neuroethics Account Res 2008 15 283 302 18972267 10.1080/08989620802388762

